# Exploratory Compatibility Regularity of Traditional Chinese Medicine on Osteoarthritis Treatment: A Data Mining and Random Walk-Based Identification

**DOI:** 10.1155/2021/2361512

**Published:** 2021-11-22

**Authors:** Qiao Zhou, Jian Liu, Ling Xin, Yanyan Fang, Lei Wan, Dan Huang, Jinchen Guo, Jianting Wen, Bing Wang

**Affiliations:** ^1^Institute of Rheumatism Prevention and Treatment of Traditional Chinese Medicine, Anhui Academy of Chinese Medicine Sciences, Hefei 230031, China; ^2^Geriatrics Department, The Second Affiliated Hospital, Anhui University of Chinese Medicine, Hefei 230061, China; ^3^Department of Rheumatism Immunity, The First Affiliated Hospital, Anhui University of Chinese Medicine, Hefei 230031, China; ^4^School of Electrical & Informatics Engineering, Anhui University of Technology, Ma'anshan 243002, China

## Abstract

Osteoarthritis (OA) is a degressive and complex disease which is a growing public health problem on a global scale. On basis of an in-house database consisting of clinical records of 13,083 OA patients, the Traditional Chinese Medicine (TCM) was divided into 4 categories of medicines on the basis of the curative properties of herbs. Due to the lack of depth and internal relationship in the calculation results of TCM compatibility law data mining methods such as statistics and frequency analysis, we use a variety of multidimensional complex network methods that can efficaciously find the compatibility law of TCM, including similarity measure, graphical visualization of network diagram, random walking, and propensity score methods. We summarize common couplet medicines utilized for the treatment of osteoarthritis. The similarity measure method was used to investigate the commonly used drugs for the treatment of osteoarthritis. The method of association rule analysis is used to recognize the compatibility between the components. On basis of the propensity score methods, the evaluation displayed that, compared with single drug, the drug group increased ESR, CRP, C3, C4, IgG, and IgA more efficiently. Concluding, a random walk model was constructed to assess drug efficacy. After applying a random walk model, while revealing the compatibility among different components of TCM, their therapeutic efficacy against OA is analyzed. We obtained four groups of drug combination clusters by similarity measure and 11 pairs of highly connected drugs by association rules, which are cardinal drug combinations in the prescription for the treatment of OA. We also found that different traditional drug pairs were associated with different laboratory indexes, and drug combinations could better optimize laboratory indexes. This study presented that the TCM constituents complement one another. Besides, the therapeutic effects resulting from a variety of combinations of these constituents are quite different.

## 1. Introduction

As a chronic progressive joint disease, osteoarthritis inflicts harm to the physical and mental health of the middle-aged and the elderly patients [1]. Pathologically, OA is characterized by simultaneous catabolic and anabolic processes which cause changes in all joint tissues. Chondrogenic degeneration, ectopic bone formation, subchondral osteosclerosis, ligament and meniscus injuries, and synovial injury in the articular cavity are the main disease markers [2]. Most of the methods remedying OA of Western medicine have their special shortcomings [3]. TCM can prevent and treat osteoarthritis through multiple levels, multiple targets, and multiple ways by the use of holistic view. What is more, traditional Chinese medicine has proved its advantages in certain aspects such as reducing the side effects of drugs, reversing drug resistance, and enhancing the quality of life and survival rate of patients [4].

Chinese medicine prescription is the treatment of a certain disease by the clinician under the guidance of the theory of Chinese medicine through a reasonable combination of medicines to maximize the strengths and avoid weaknesses, adjust its bias, reduce its toxicity, enhance or change its original effect, and eliminate or alleviate its disadvantages to the human body. The pharmacological and pharmacodynamic relationship between herbs is considered to be the compatibility of Chinese medicine treatment. The law of compatibility of TCM is a key issue in the study of Chinese medicine prescription. It is the core aim of Chinese medicine prescription research to excavate the law of compatibility and clarify the scientific connotation of compatibility. As an efficacious method and significant technique to grope for traditional Chinese medicine, data mining method has formed a set of standardized research models and systems in the mining of compatibility law of TCM, which can better reveal the compatibility relationship between drugs [5].

This research is designed to explore the compatibility regularity and clinical therapeutic role of TCM in the treatment of osteoarthritis. We use a variety of data mining tools, such as cluster analysis, association rules, graphical visualization of network graphs, propensity scoring methods, and random walk models to analyze clinical OA data. The data mining method can analyze the frequency of herbs, the rule of formulation, and the change of formulation pattern obtained from the knowledge graph.

## 2. Materials and Methods

### 2.1. Materials

The inpatient data were collected for inpatients who were OA inpatients between July 2009 and March 2021 in the Department of Rheumatology and Immunology of the First Affiliated Hospital of Anhui University of Chinese Medicine. The dataset includes the use of Chinese herbal medicine, Huangqin Qingre Chubi capsules prescription preparation, Furong ointment, and disease-associated laboratory indices such as the inflammatory markers CRP and ESR and the immune indexes IgA, IgM, IgG, C3, and C4. The Ethics Committee of the First Affiliated Hospital of Anhui University of Chinese Medicine approved the study protocol. There were 13,776 patients with OA examined, including 13,083 with the treatment of Chinese herbal medicine. All patients were divided into a control group (Chinese herbal medicine alone) and an experimental group (Chinese herbal medicine plus Huangqin Qingre Chubi capsule/Furong ointment prescription preparation). The control group had 7,119 cases, and the experimental group had 5,964 cases.

### 2.2. Methods

#### 2.2.1. Similarity Measure

We used Chinese herbal medicine as a variable, and we set it as 1 if used and 0 if not used. The systematic cluster analysis method is used to study the compatibility of Chinese herbal medicine by SPSS 22.0 (IBM Corp., Armonk, NY, USA). We treat each herb as a point and calculate the distance between the points. The ones that are close to each other fall into one category, and the ones that are far away fall into another category. The similarity of Chinese herbs was calculated using the mean Euclidean measure [6]. Setting *x*_*i*_ and *y*_*i*_ (*i* = 1, 2,… *n*) as the continuous points in the two-dimensional metric space of signal *x* and signal *y*, the Euclidean distance of x and *y* can be defined as follows [7]:

(1) $$\begin{array}{c} d\left(x,y\right)=\sqrt{\sum_{i=1}^{n}{\left(x_{i}-y_{i}\right)}^{2}}. \end{array}$$

In the above equation, *x*_*i*_ and *y*_*i*_ are the *i*th sampling data points of signal *x* and signal *y*, respectively, and *n* is the total number of sampling points. An analysis of the equation demonstrates that the higher the similarity between signal *x* and signal *y* is, the smaller the value of the Euclidean distance *d*(*x*, *y*) is. On the contrary, the lower the similarity between signal *x* and signal *y* is, the larger the value of the Euclidean distance *d*(*x*, *y*) is.

#### 2.2.2. Association Rules

*(1) Apriori Algorithm*. 1 was used to indicate the application of Chinese herbal medicine or indicators, while no use was indicated by 0. The interdependency of Chinese herbs medicine was identified by Apriori module in SPSS Clementine v.11.1 (IBM Corp., Armonk, NY, USA). We set the minimum support to 60%, confidence to 80%, and improvement to >1. Nowadays, the data mining technology of association rules is mainly on basis of Apriori algorithm, and its key optimization is to find all frequent itemsets in the trade database [8]. As a pattern of the form *X* ⟶ *Y*, an association rule means that the presence of the itemsets *X* is associated with *Y* in transactions [9]. The support degree, confidence degree, and Lift degree of an association rule between X and *Y* are, respectively, as follows [10]:

(2) $$\begin{array}{c} \text{Support}\left(X,Y\right)=P\left(XY\right)=\frac{\text{number}\left(XY\right)}{\text{num}\left(\text{AllSamples}\right)},\\ \text{Confidence}\left(X⇐Y\right)=P\left(X|Y\right)=\frac{P\left(XY\right)}{P\left(Y\right)},\\ \text{Lift}\left(X⟶Y\right)=P\left(Y|X\right)|P\left(Y\right). \end{array}$$

*(2) Graphical Visualization of Network Diagram*. We use the SPSS Clementine v.11.1 (IBM Corp., Armonk, NY, USA) “network” node to analyze Chinese medicine. Set the threshold to be absolute, strong links are thicker, and the maximum number of links that can be displayed is 80. The upper limit of weak links is 35, the lower limit of strong links is 100, and the link size shows continuous changes. The thick, thin, and dashed lines indicate the strength of the links between drugs to generate an overall network diagram.

#### 2.2.3. Propensity Score Methods

Propensity score approaches, extensively applied to regulate for confounding in observational researches with dichotomous treatment modalities, mimic the intended roles of randomization by the balance of measured baseline covariates across treatment groups [11]. *Z* means treatment allocation, *Y* the continuous outcome, and the baseline covariates are displayed as *X*  =  (*X*_1_,…, *X*_*p*_); the propensity score is defined as [12]

(3) $$\begin{array}{c} e\left(X\right)=P\left(Z=1|X\right). \end{array}$$

As the treatment index Z is binary and it is supposed that the logistic regression is parametrized by *α* = (*α*_0_, *α*_1_,…, *α*_*p*_)^⊤^,

(4) $$\begin{array}{c} \log\left\{\frac{\left.e\left(X\right)\right)}{\left(1-e\left(X\right)\right)}\right\}-X^{⊤}\alpha . \end{array}$$

For every participant indexed by subscript *i*, it is possible to estimate a probability in the treatment or control arm, given the baseline features from the fitted propensity score model as

(5) $$\begin{array}{c} {\hat{e}}_{i}=\hat{e}\left(X_{i}\right)=\frac{\exp\left(X_{i}^{⊤}\hat{\alpha }\right)}{1+\exp\left(X_{i}^{⊤}\hat{\alpha }\right)}. \end{array}$$

**Figure 1:**
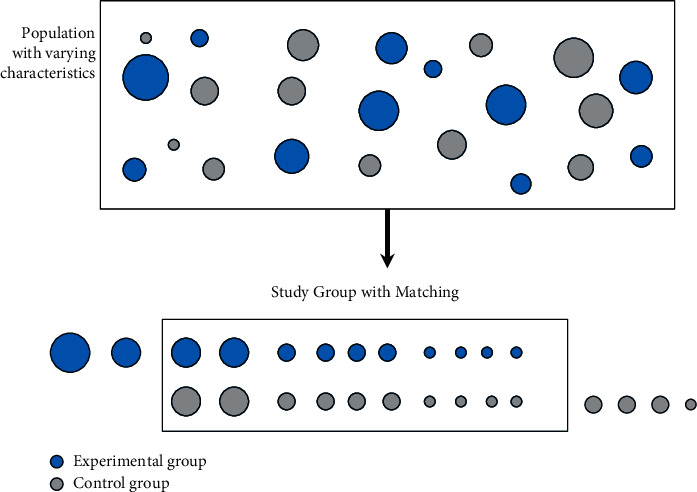
Diagrammatic sketch of propensity score methods.

The schematic diagram of propensity matching is shown in Figure 1.

#### 2.2.4. Random Walking

The evaluation of the laboratory indices random walking model is realized through the ORACLE 10 g tool.

Random walk was first put forward by Pearson in 1905 [13]. The walk upward movement is (*u*(*i*) = +1) and the downward movement is down (*u*(*i*) = −1) by one-unit length (*u*) for each step *i*. Consequently, the random walk ultimately stimulates the quantification of this correlation by calculating the “net displacement” (*y*) of the walker after one step, which is the sum of the unit steps *u*(*i*)*f* of each step *i*[14]:

(6) $$\begin{array}{c} \gamma \left(l\right)=\sum_{i=1}^{1}u\left(i\right). \end{array}$$

The root mean square fluctuation *F*(*l*) about the average of the displacement is an important statistical quantity characteristic. *F*(*l*) is defined as the difference between the average of the square and the square of the average,

(7a) $$\begin{array}{c} F^{2}\left(l\right)=\bar{\left[\Delta \gamma \left(l\right)\right.-\bar{{\left.\Delta y\left(l\right)\right]}^{2}}}=\bar{{\left[\Delta y\left(l\right)\right]}^{2}}-{\left[\bar{\Delta y\left(l\right)}\right]}^{2}, \end{array}$$

of a quantity Δ*y*(*l*) defined by

(7b) $$\begin{array}{c} \Delta \gamma \left(l\right)=\gamma \left(l_{0}+l\right)-\gamma \left(l_{0}\right). \end{array}$$

We can comprehend that equation (1) walks a set of calipers with a fixed distance gauge, equation (2) sequentially moves the starting point from *l*_0_=1 to *l*_0_=2 and so on, equation (6) calculates the quantity Δ*y*(*l*) and its square for each *l*_0_, and equations (7a) and (7b) average all calculated quantities to obtain the following equation:

(8) $$\begin{array}{c} F^{2}\left(l\right)∼l^{a},\;\text{with }\alpha \neq \frac{1}{2}. \end{array}$$

### 2.3. Statistical Processing

The analysis of all data was made by SPSS v.22.0 (IBM Corp., Armonk, NY, USA). The operation of a nonparametric test on two associated samples was made for the control and experimental groups before and after treatment. Variations in characteristics between groups were analyzed using the Mann-Whitney rank-sum test or *χ*^2^ tests. Variations were recognized to be statistically significant at *P* < 0.05.

## 3. Result

### 3.1. Administration of Chinese Herbal Medicine in OA Treatment

There were 13,776 patients with OA researched, among whom 13,083 were cured with Chinese herbal medicine. Every OA patient took 1 prescription of Chinese herbal medicine with >420 kinds. The first 20 Chinese herbal medicines were separated into 4 categories in the light of their potent effect: *Poria, Radix Glycyrrhizae, Pericarpium Citri Reticulatae, Semen Coicis*, and *Rhizoma Dioscoreae Oppositae* to invigorate spleen for eliminating dampness; *Flos Carthami, Radix Salviae Miltiorrhizae, Semen Persicae, Caulis Spatholobi*, and *Radix Achyranthis Bidentatae* to promote blood circulation to remove meridian obstruction; *Radix et Rhizoma Clematidis Chinensis, Herba Siegesbeckiae, Radix Angelicae Biserratae, Herba Gaultheriae Grenulatae*, and *Herba Lycopodii Japonici* to dispel wind and eliminate dampness; and *Herba Taraxaci Mongolici, Herba Hedyotis, Plantago asiatica* L.*, Cortex Phellodendri Amurensis*, and *Radix Scutellariae Baicalensis* to clear away heat and toxic materials. On the basis of five flavors, sweet taste is supervised up to a maximum of 59,458 times, while bitter taste is given at the most 69,650 times. According to tropism of taste of Chinese herbal medicine, there are 44,470 uses of Chinese herbal medicine belonging to the spleen meridian maximally (Table 1).

### 3.2. Similarity Measure of Chinese Herbal Medicine in the Treatment of Osteoarthritis

**Figure 2:**
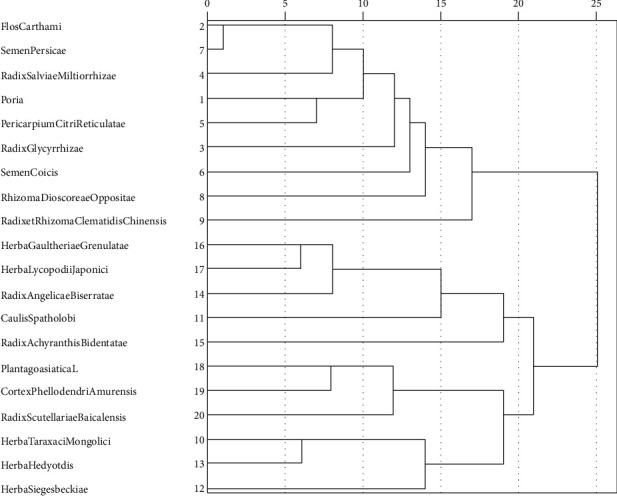
Similarity measure of Chinese herbal medicine treatment of osteoarthritis. Note: Euclidean distance = 15 o'clock; Chinese medicine is separated into four groups. Neither *Radix et Rhizoma Clematidis Chinensis* nor *Radix Achyranthis Bidentatae* is included in other taxa.

The systematic clustering method for cluster analysis of 20 traditional Chinese medicines for treating osteoarthritis is adopted. When the Euclidean distance is 15, the following four groups of drug combination clusters can be obtained (the other 2 drugs are invalid clusters) (Figure 2).

  Set1: *Flos Carthami, Radix Salviae Miltiorrhizae, Poria, Radix Glycyrrhizae, Semen Coicis, Pericarpium Citri Reticulatae, Rhizoma Dioscoreae Oppositae,* and *Semen Persicae*.
  Set2: *Herba Gaultheriae Grenulatae*, *Herba Lycopodii Japonici*, *Radix Angelicae Biserratae*, and *Caulis Spatholobi*.
  Set3: *Plantago asiatica* L.*, Cortex Phellodendri Amurensis,* and *Radix Scutellariae Baicalensis.*
  Set4: *Herba Taraxaci Mongolici, Herba Hedyotis,* and *Herba Siegesbeckiae.*

### 3.3. Analysis of Association Rules of Traditional Chinese Medicine or Immune-Inflammatory Indices in the Treatment of Osteoarthritis

**Figure 3:**
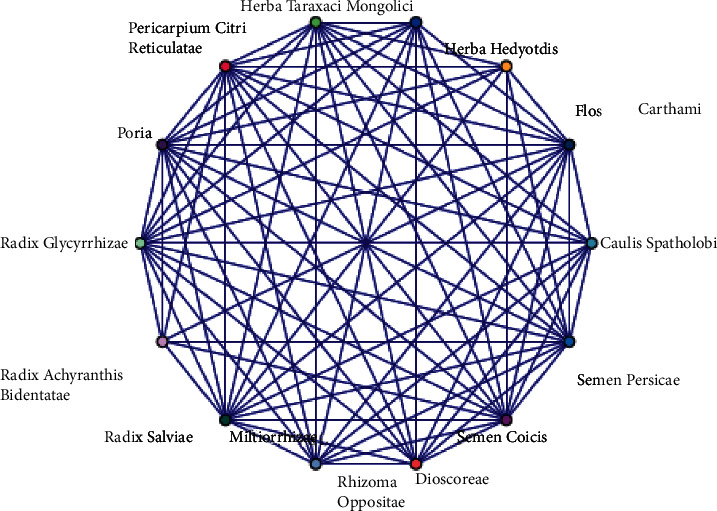
Graphical visualization of network diagram of Chinese herbal medicine in OA treatment.

The compatibility of the two TCM is to increase efficiency and reduce toxicity. The compatibility mode of the prescription is further tested. We analyze the correlation degree of core Chinese medicine, where the minimum support is set to 70%, and the minimum confidence is set to 80%. The lift is > 1, *P* ≤ 0.001. According to the analysis results of association rules, the higher the promotion (gain), the stronger the correlation. We define each traditional Chinese medicine as an itemset. Finally, we secured 11 pairs of highly connected drugs. They are cardinal drug combinations in the prescription for the treatment of OA. The improvement degree of all drug combinations is greater than 1.0, indicating that these drug combinations are statistically significant (Table 2). In addition, by constructing an association network diagram of high-frequency drugs, it clearly and intuitively reflects the degree of association between drugs. *Herba Taraxaci Mongolici, Herba Hedyotis, Flos Carthami, Caulis Spatholobi, Semen Persicae, Semen Coicis, Rhizoma Dioscoreae Oppositae, Radix Salviae Miltiorrhizae, Radix Achyranthis Bidentatae, Radix Glycyrrhizae,* and *Poria* have strong correlation in node degree. They often appear in pairs and are the core drug combination (Figure 3). Simultaneously, the minimum support is set to 60% and the minimum confidence to 80%; after the analysis of the Apriori module, each item is ranked with the highest confidence level. We conduct correlation analysis on drugs and immune-inflammatory indices to analyze the relationship between immune-inflammatory indices optimization and Chinese herbs compatibility. We found that *Herba Taraxaci Mongolici, Flos Carthami,* and *Semen Coicis* are related to inflammatory index, such as ESR and CRP. *Semen Persicae, Poria, Radix Glycyrrhizae,* and *Radix Salviae Miltiorrhizae* are related to immune index, for instance, IgM, IgA, and IgG. *Radix Glycyrrhizae* and *Pericarpium Citri Reticulatae* are bounded up with C4 and C3. The improvement degree of the above correlation results is > 1 (Table 3).

### 3.4. Improvement of Immune-Inflammatory Indices

First, we assembled one-to-one propensity score methods matching the clinical background between the two groups to minimize the imbalance from measured baseline covariates. The matched groups were balanced by age, sex, BMI, length of stay, and underlying diseases (coronary heart disease, hypertension, cerebral infarction, diabetes, chronic gastritis, anemia, osteoporosis, and fatty liver) (Table 4). One-to-one nearest neighbour caliper matching was used to match patients based on the propensity score using a caliper equal to 0.2 of the SD of the logit of the propensity score. The age, sex, BMI, LOS, and underlying diseases (coronary heart disease, hypertension, cerebral infarction, diabetes, and osteoporosis) were significantly different between the experimental group and the control group but were comparable between the matched experimental group and the matched control group after propensity score matching. The immune-inflammatory indices between the matched experimental group and the matched control group were also comparable. After that, by comparison with those before treatment, ESR, IgA, IgG, CRP, IgM, C3, and C4 were reduced greatly in both groups after treatment. After treatment, ESR, IgA, CRP, IgG, C3, and C4 reduced greatly in the experimental group compared to the control group (Table 5).

### 3.5. Evaluation of Immune-Inflammatory Indices by Random Walking Model

**Figure 4:**
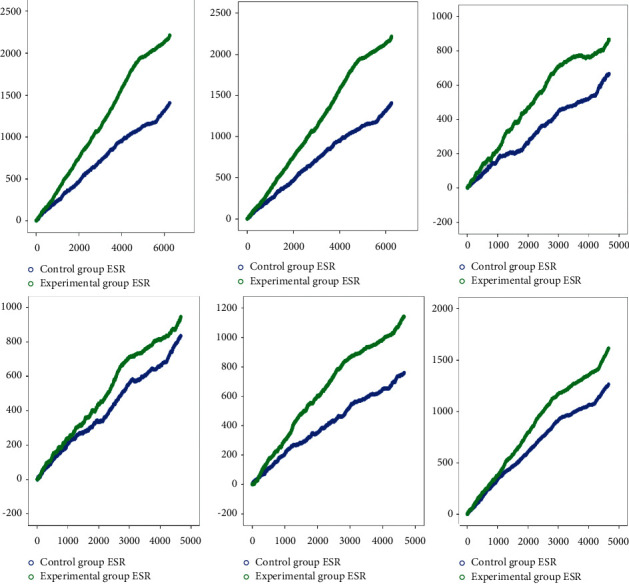
Random walking model of immune-inflammatory indices in OA patients. Note: green line represents the experimental group. Blue line represents the control group. The length of the horizontal line increases with the increase in the number of walking steps. The height of the vertical line increases with intervention efficacy and response.

The ESR of the control group had a total of 6253 comprehensive evaluation records. The clinical significance is that, for every increase in the integrated index of the patient, 9.26 steps need to be walked, or, every step forward, the comprehensive improvement rate is 22.60%. There are a total of 6883 comprehensive evaluation records of ESR in the experimental group. The clinical significance is that, for every increase in the integrated index of the patient, 5.11 steps need to be walked, or, every step forward, the integrated improvement rate is 36.20%. There were 6,933 and 7,603 integrated evaluation records for CRP in both groups. The patient improvement indexes in both groups were 0.276 and 0.426, respectively. With regard to the clinical significance, for every increase in the integrated index, the patients had to walk 7.28 and 4.18 steps. There are 4676 integrated evaluation records of IgA in the control group. The clinical significance is that every time the patient's comprehensive index improves by one point, they need to walk 17.33 steps, or, every step forward, the comprehensive improvement rate is 14.20%. There are 4794 comprehensive evaluation records of IgA in the experimental group. The clinical significance is that every time the patient's comprehensive index improves by one point, they need to walk 12.05 steps, or, every step forward, the comprehensive improvement rate is 18.40%. There were 4,675 and 4,800 integrated evaluation records for IgG in both groups. The patient improvement indexes in both groups were 0.179 and 0.204. The clinical significance was that, for every increase in the integrated index, the patients had to walk 13.82 and 10.86 steps. There are 4670 integrated evaluation records for C3 in the control group. The clinical significance is that every time the comprehensive index of the patient improves by one point, they need to walk 15.17 steps, or, every step forward, the comprehensive improvement rate is 16.30%. There are 4796 comprehensive evaluation records for patients in the treatment group's C3. The clinical significance is that every time the comprehensive index of the patient improves by one point, they need to walk 9.07 steps, or, every step forward, the comprehensive improvement rate is 24.40%. There were 4,670 and 4,796 integrated evaluation records for C4 in both groups. The patient improvement indexes in both groups were 0.270 and 0.353. With regard to the clinical significance, for every increase in the integrated index, the patients had to walk 9.12 and 6.28 steps (Table 6 and Figure 4).

## 4. Discussion and Conclusion

Clustering is unsupervised learning and separated the data from groups (call as clusters) on account of their similar attributes. Apriori algorithm is a typical Boolean association rule frequent itemset algorithm [15]. The long-term correlation and mathematical probability theory of the random walking model are similar to the development of human diseases. Each symptom of the disease is affected by many factors such as the patient's self-perception, the body's ability to resist disease, and treatment measures. Whether the random walk model has long-term correlation, whose direct meaning is whether the index system is effective, is of great significance to the establishment of a comprehensive index system that is widely recognized and effective in Chinese medicine clinics.

OA is regarded as a kind of “arthralgia syndrome” in traditional Chinese medicine theory [16]. Traditional Chinese medicine treatment can effectively improve pain, dysfunction, and other symptoms and reduce the recurrence rate [17]. Herbal therapy is on the basis of the syndrome differentiation for the sake of satisfy personalized needs of different patients. Despite the use of single herb, physicians love herbal formulas, as well as complex mixtures of numerous herbs with abundant therapeutic compounds to maximize the curative effects and minimize the toxicity or adverse effects by interplay of different herbs [18]. As the painful obstruction, arthralgia syndrome signifies that either the limbs or the joints are subjected to pain and malfunction. The evil influence such as wind, cold, damp, and heat invades the physical organism, blocking the meridians and consuming the Promordial Qi and blood, which will make the joints painful, swollen, stiff and, in severe cases, deformed. Optimal administration of OA appeals for individual methods on the basis of various constitutions of human body. The individually integrated TCM approach in this research has been generated by clinical experience from the First Affiliated Hospital [19]. The approach comprises a fundamental treatment of orally administered Chinese herbal drugs and herbal patch on the basis of the severity of the patient symptoms.

Here, cluster and association rule analyses, graphical visualization of network diagram, propensity score methods, and random walking model data mining were applied to discriminate Chinese herbal medicine for OA treatment, compatible combinations, and significant therapeutic efficacy against OA.

In the 13,083 Chinese herbal prescriptions, 426 types of herbs were applied to OA treatment. In the light of the nature, flavor, meridian tropism, and main efficacy of medicines, it was sensible to divide these into four categories tally with the principles of TCM, as shown in Table 1. The frequency of use of invigorating spleen for eliminating dampness herbs was 47,424, which was the highest. The most commonly used single-flavor medicines are *Poria, Flos Carthami, Radix Glycyrrhizae, Radix Salviae Miltiorrhizae, Pericarpium Citri Reticulatae*, and so forth. Classified according to five flavors, sweetness and bitterness are the two most frequently used. Within the four classes of Chinese herbal medicine, the spleen meridian was used 44,470 times, sweet taste was applied 59,458 times, and bitter taste was applied 69,650 times. Sweet taste can replenish energy and tonify the spleen, while bitter taste is used to dispel dampness, on account of traditional Chinese medicine ideology. Both can verify that OA pathogenesis originates mainly from spleen deficiency and dampness. The development of muscle and its function are closely related to the function of spleen transportation. Based on modern pharmacology study, spleen-invigorating Chinese medicine can increase immune capacity, improve learning and memory function, and control the endocrine function of patients with impaired disease [20]. Through data mining technology, the high-frequency herbs and Chinese herbs compatibility for the treatment of osteoarthritis are summarized, and the drug characteristics and prescription rules of osteoarthritis are scientifically and objectively revealed. At length, we come to the conclusion that the regularity of traditional Chinese medicine in the treatment of osteoarthritis is invigorating spleen for removing dampness, promoting blood circulation and removing blood stasis, expelling wind and removing dampness, and clearing away heat and toxic materials, which can be used for reference in the treatment of clinical osteoarthritis.

Through cluster analysis, we found that high-frequency Chinese medicine combinations commonly used in this disease can be divided into four categories (Figure 2). The first group of Chinese medicines is to invigorate the spleen, dispel dampness, and promote blood circulation. The second kind of Chinese medicine expels wind and dredges collaterals. The third kind of Chinese medicine clears away heat and detoxifies. The fourth kind of traditional Chinese medicine is to clear away heat and detoxify and relieve collaterals.

According to the set of principles for drug association analysis, there were a total of 11 rules involving couplet medicines resulting. There are some interesting association rules that are usually used by ancient and modern physicians for combinations of couplet medicines. The couplet medicine of invigorating the spleen medicinal is *Pericarpium Citri Reticulata-Poria* and *Radix Glycyrrhizae-Poria*, while blood-activating medicinal is *Radix Salviae Miltiorrhizae-Flos Carthami* and *Flos Carthami-Semen Persicae,* for instance. We find that the obtained association rules invigorating the spleen, dispelling dampness, promoting blood circulation, and clearing away heat and detoxifying treatments have concomitant uses. Based on the relationship rule mining, synergistic herbal groups could be derived; nevertheless, it is necessary to further analyze the associated mechanism (Table 2 and Figure 3). At the same time, there is a strong correlation between the compatibility of traditional herbal medicine and the optimization of immune-inflammatory indices (Table 3). *Herba Taraxaci Mongolici, Flos Carthami,* and *Semen Coicis* are related to inflammatory index, such as ESR and CRP. The new research demonstrated that taraxasterol has the in vitro anti-inflammatory effect, which is one of the chief active components isolated from *Herba Taraxaci Mongolici* [21]. *Flos Carthami* [22] and *Semen Coicis* [23] are perhaps connected to its antioxidation and anti-inflammatory properties. *Semen Persicae, Poria, Radix Glycyrrhizae,* and *Radix Salviae Miltiorrhizae* are related to immune index, for instance, IgM, IgA, and IgG. *Radix Glycyrrhizae and Pericarpium Citri Reticulatae* are bounded up with C4 and C3. *Poria* [24] is commonly used as a tonic and antiaging traditional Chinese medicine, which is traditionally used in combination with other TCM to enhance immunity. *Radix Glycyrrhizae Polysaccharide*, one of the chief bioactive components in *Radix Glycyrrhizae*, has been reported to participate in regulation of immunity and phagocytosis, as well as anticomplement [25]. Volatile oils and flavonoids in *Pericarpium Citri Reticulatae* are considered to be major components, which act alone or jointly to combat inflammatory responses and lipid peroxidation, followed by attenuating immunological reaction [26]. To conclude, the therapeutic effects resulting from combinations of couplet medicines are quite different.

The generation of Chinese formulas with various herbs is not random. In this research, data mining technology was used to determine the rules for the application of Chinese herbal medicine in OA treatment in our hospital, in order to test the efficacy of Chinese herbal medicine in OA treatment. As a convenient prescription hospital preparation of Anhui Traditional Chinese Medicine Hospital, Huangqin Qingre Chubi capsule (Anhui medicine No. ZL201110095718.X) consists of *Radix Scutellariae Baicalensis*, *Radix et Rhizoma Clematidis Chinensis*, *Semen Coicis*, and *Semen Persicae*. It has the function of invigorating spleen and removing dampness and clearing heat and removing arthralgia with long history in clinical OA treatment. As a prescription preparation of our hospital, Furong ointment has the effect of clearing heat, detoxifying swelling, cooling blood, and relieving pain.

The variations in the values of immune-inflammatory indices were analyzed to evaluate the efficacy. Before that, we used propensity scoring method to match the baseline characteristics and immune-inflammatory indices of the two groups to reduce research bias. As you can see, the propensity score matching resulted in 4438 pairs of patients from the experimental group and the control group. Unlike the results before adopting propensity score matching, there were no significant differences between both groups in terms of age, sex, BMI, LOS, and underlying diseases (coronary heart disease, hypertension, cerebral infarction, diabetes, and osteoporosis) (Table 4) (*P* > 0.05). However, inevitable imbalance persisted in chronic gastritis, anemia, and fatty liver. After applying propensity score matching, the immune-inflammatory indices were balanced between the two groups, and there was no significant difference (*P* > 0.05) (Table 4). According to the distribution rules, there were 7,119 patients in the control group and 5,964 patients in the experimental group. The statistical results show that, compared with single drug, the drug combination improved ESR, C3, IgA, CRP, IgG, and C4 more effectively (Table 5). Therefore, strong therapeutic efficacy of Chinese herbal decoctions integrated with prescription preparations was found. A random walking model was applied to assess the immune-inflammatory indices of both groups of OA patients. The patient's immune-inflammatory indices refer to the long-term relationship between the changes of ESR, IgA, IgG, C3, CRP, and C4 and the intervention measures the patient receives, which means that the treatment measures the patient receives affect the changes of the patient's immune inflammation index. The treatment group's ESR, IgA, CRP, C3, C4, and IgG are better than the control group's in terms of the maximum random fluctuation, the positive growth rate of walking, the increase rate of the comprehensive evaluation index, the comprehensive improvement rate, the number of records of the comprehensive evaluation index, and the expected improvement value. But the improvement of IgM is not obvious. There is a long-term correlation between the comprehensive evaluation indexes and intervention measures of the two groups of patients, and the improvement effect of indexes of the experimental group is better than that of the control group (Table 6 and Figure 4).

The Chinese medicine is through the classification of the causes of OA, symptomatic administration, and the use of appropriate methods of treatment [27]. As for the diagnosis and treatment model based on diagnostic personality and diagnostic integration, TCM medical records provide good evidence for TCM evidence-based practice, which reflects the comprehensive application of TCM principles, methods, formulas, and drugs rather than the comprehensive application of TCM. It is not only the true record of medical activities but also the reflection of the physician's clinical experience and thought process. Data mining, also known as knowledge discovery, is the task of discovering laws or patterns hidden in a large amount of data, which can bring improvement and further development of TCM academic technology [28]. However, Chinese medicine discusses and studies human body from the whole and function level, while data mining based on data induction and machine learning is limited to exploring the surface statistical law and lacks the discussion on the internal mechanism of the system. On the premise of guaranteeing the integrity of the data, therapeutic efficacy is difficult to be fully verified because of the complexity of the inpatient medical records, including the incomplete and unquantifiable information in the data. Beyond that, Chinese medicine studies human body from the whole and function level, but the data mining based on data induction and machine learning is limited to exploring the surface statistical law and lacks the discussion of the internal mechanism of the system.

In conclusion, we built a random walking model, which is based on laboratory indicators and combined with cluster analysis, association rules, graphical visualization of network diagram, and propensity score methods. We found that Chinese medicine compatibility can improve the immune-inflammatory indices of patients suffering from OA. Different groups of these drugs bring about various effects in OA treatment, which provide experience for clinical treatment of OA.

## Figures and Tables

**Table 1:** Application of Chinese herbal medicine in OA treatment.

Category	Herb	Number	Nature and taste	Meridian tropism
Invigorating spleen for eliminating dampness	*Poria*	10376	Sweet, flat, light	Spleen, kidney
	*Radix Glycyrrhizae*	9789	Gump, flat	Heart, lung, stomach, spleen
	*Pericarpium Citri Reticulatae*	9625	Pungent, warm, bitter	Spleen
	*Semen Coicis*	9044	Sweet, light	Spleen, stomach
	*Rhizoma Dioscoreae Oppositae*	8590	Sweet, flat	Spleen, lung, kidney

Removing meridian obstruction by promoting blood circulation	*Flos Carthami*	10100	Pungent, warm	Heart, liver
	*Radix Salviae Miltiorrhizae*	9769	Bitter, slightly cold	Heart, liver
	*Semen Persicae*	8593	Bitter, flat, sweet	Heart, liver
	*Caulis Spatholobi*	6622	Bitter, sweet, warm	Liver, kidney
	*Radix Achyranthis Bidentatae*	4284	Bitter, sweet, sour, flat	Liver, kidney

Dispelling wind and eliminating dampness	*Radix et Rhizoma Clematidis Chinensis*	7743	Pungent, salt, warm	Bladder
	*Herba Siegesbeckiae*	5511	Bitter, bitter, slightly cold	Liver, kidney
	*Radix Angelicae Biserratae*	4395	Pungent, bitter, warm	Kidney, bladder
	*Herba Gaultheriae Grenulatae*	4127	Sweet, pungent	Lung, liver
	*Herba Lycopodii Japonici*	3606	Bitter, pungent, warm	Liver, spleen, kidney

Eliminating heat and toxic materials	*Herba Taraxaci Mongolici*	7288	Bitter, slightly cold, sweet	Liver, stomach
	*Herba Hedyotis*	4661	Bitter, slightly cold, sweet	Stomach, larger intestine
	*Plantago asiatica* L.	3034	Sweet, slightly cold	Liver, kidney, lung
	*Cortex Phellodendri Amurensis*	2832	Bitter	Kidney, bladder
	*Radix Scutellariae Baicalensis*	2464	Bitter	Lung, gallbladder, spleen

**Table 2:** Association of Chinese herbal medicine in OA treatment.

Items (LHS ⇒ RHS)	Support (%)	Confidence (%)	Lift	*P* value
*{Pericarpium Citri Reticulatae}* ⇒ *{Poria}*	73.57	87.71	1.11	≤0.001
*{Radix Salviae Miltiorrhizae}* ⇒ *{Flos Carthami}*	74.67	85.38	1.11	≤0.001
*{Radix Salviae Miltiorrhizae}* ⇒ *{Poria}*	74.67	85.02	1.07	≤0.001
*{Flos Carthami}* ⇒ *{Semen Persicae}*	77.20	83.93	1.28	≤0.001
*{Radix Glycyrrhizae}* ⇒ *{Poria}*	74.82	83.69	1.05	≤0.001
*{Pericarpium Citri Reticulatae}* ⇒ *{Flos Carthami}*	73.56	83.28	1.08	≤0.001
*{Flos Carthami}* ⇒ *{Poria}*	77.20	82.87	1.04	≤0.001
*{Pericarpium Citri Reticulatae}* ⇒ *{Radix Salviae Miltiorrhizae}*	73.57	82.75	1.11	≤0.001
*{Flos Carthami}* ⇒ *{Radix Salviae Miltiorrhizae}*	77.20	82.58	1.11	≤0.001
*{Radix Salviae Miltiorrhizae}* ⇒ *{Pericarpium Citri Reticulatae}*	74.67	81.53	1.11	≤0.001
*{Poria}* ⇒ *{Pericarpium Citri Reticulatae}*	79.31	81.36	1.11	≤0.001

Values are % degrees of relevancy.

**Table 3:** Association of Chinese herbal medicine and immune-inflammatory indices in OA treatment.

Items (LHS ⇒ RHS)	Support	Confidence	Lift	*P* value
{*Herba Taraxaci Mongolici* and *Flos Carthami*} ⇒ {ESR}	60.52	82.86	1.02	≤0.001
{*Herba Taraxaci Mongolici* and *Semen Coicis*} ⇒ {CRP}	61.64	87.46	1.02	≤0.001
{*Semen Persicae* and *Poria* } ⇒ {IgA}	61.82	83.50	1.02	≤0.001
{*Radix Glycyrrhizae* and *Poria*} ⇒ {IgM}	64.81	75.25	1.03	≤0.001
{*Radix Glycyrrhizae* and *Radix Salviae Miltiorrhizae*} ⇒ {IgG}	63.11	99.89	1.00	≤0.001
{*Radix Glycyrrhizae* and *Pericarpium Citri Reticulatae*} ⇒ {C3}	62.32	85.85	1.01	≤0.001
{*Radix Glycyrrhizae* and *Pericarpium Citri Reticulatae*} ⇒ {C4}	68.73	89.82	1.00	≤0.001

Values are % degrees of relevancy.

**Table 4:** Baseline characteristics of all patients.

Variables		Before matching			After matching		
		Control group (*n* = 7,119)	Experimental group (*n* = 5,964)	*P* value	Control group (*n* = 4,438)	Experimental group (*n* = 4,438)	*P* value
Demographics	Age/year, mean ± SD	58.56 ± 13.71	62.75 ± 12.88	<0.001	60.06 ± 13.84	59.84 ± 12.82	0.068

Sex	Male, *n* (%)	1,402 (19.69)	1,582 (26.53)	<0.001	907 (20.43)	912 (20.55)	0.072
	Female, *n* (%)	5,717 (80.31)	4,382 (73.47)	<0.001	3,531 (79.56)	3,526 (79.45)	0.080

Anthropometric measurements	BMI, kg/m^2^	28.36 ± 5.60	26.56 ± 6.50	<0.001	27.12 ± 6.25	27.68 ± 6.04	0.094

Clinical characteristics	LOS, days, mean ± SD	15.05 ± 7.77	17.10 ± 9.08	<0.001	16.36 ± 8.36	16.49 ± 7.91	0.074

Underlying diseases	Coronary heart disease, *n* (%)	354 (4.97)	233 (3.91)	<0.001	189 (4.26)	183 (4.12)	0.061
	Hypertension, *n* (%)	193 (2.71)	1,243 (20.84)	<0.001	193 (4.34)	199 (4.48)	0.056
	Cerebral infarction, *n* (%)	236 (3.32)	768 (12.88)	<0.001	229 (5.16)	231 (5.21)	0.063
	Diabetes, *n* (%)	119 (1.67)	610 (10.23)	<0.001	119 (2.68)	132 (2.97)	0.051
	Chronic gastritis, *n* (%)	1,353 (19.01)	744 (12.47)	<0.001	572 (12.89)	619 (13.95)	<0.001
	Anemia, *n* (%)	207 (2.91)	167 (2.80)	<0.001	93 (2.10)	133 (3.00)	<0.001
	Osteoporosis, *n* (%)	1,861 (26.14)	1,616 (27.10)	<0.001	1,214 (27.35)	1,202 (27.08)	0.063
	Fatty liver, *n* (%)	768 (10.79)	700 (11.74)	<0.001	361 (8.13)	475 (10.70)	<0.001

Index	ESR (mm/h)	28.79 ± 15.89	14.44 ± 11.97	<0.001	30.18 ± 16.92	29.26 ± 12.84	0.112
	CRP (mg/L)	14.44 ± 7.96	27.80 ± 9.26	<0.001	14.66 ± 8.48	16.50 ± 7.12	0.124
	IgA (g/L)	2.35 ± 1.07	2.65 ± 1.25	<0.001	2.38 ± 1.09	2.41 ± 1.21	0.085
	IgM (g/L)	1.26 ± 0.67	1.23 ± 0.72	0.427	1.20 ± 0.63	1.21 ± 0.70	0.354
	IgG (g/L)	12.40 ± 3.99	15.91 ± 4.06	<0.001	12.61 ± 4.04	13.04 ± 4.14	0.214
	C3 (g/L)	83.69 ± 49.72	93.12 ± 53.61	<0.001	97.16 ± 39.22	90.01 ± 54.33	0.359
	C4 (g/L)	26.04 ± 13.44	22.27 ± 14.53	<0.001	23.43 ± 11.69	22.50 ± 14.69	0.212

Note: BMI, body mass index; LOS, length of hospital stay; ESR: erythrocyte sedimentation rate; CRP: C-reactive protein; IgA: immunoglobulin A; IgM: immunoglobulin M; IgG: immunoglobulin G; C3: complement C3; C4: complement C4.

**Table 5:** Improvement of immune-inflammatory indices.

Index	Control group		Experimental group		
	*Z*_0_	*P*_0_ value	*Z*_1_	*P*_1_ value	*P*_2_ value
ESR (mm/h)	−25.59	≤0.001	−37.85	≤0.001	≤0.001
CRP (mg/L)	−33.98	≤0.001	−48.04	≤0.001	≤0.001
IgA (g/L)	−14.54	≤0.012	−19.10	≤0.001	≤0.001
IgM (g/L)	−2.50	≤0.001	−5.25	≤0.001	0.242
IgG (g/L)	−17.92	≤0.001	−66.13	≤0.001	≤0.001
C3 (g/L)	−15.09	≤0.001	−22.79	≤0.001	≤0.001
C4 (g/L)	−24.81	≤0.001	−30.30	≤0.001	≤0.001

Note: ESR: erythrocyte sedimentation rate; CRP: C-reactive protein; IgA: immunoglobulin A; IgM: immunoglobulin M; IgG: immunoglobulin G; C3: complement C3; C4: complement C4. *Z*_0_ represents the standardized test statistics before and after treatment in the control group. *P*_0_ represents the comparison of the control group before and after treatment. *Z*_1_ represents the standardized test statistics before and after treatment in the experimental group. *P*_1_ represents the comparison of the experimental group before and after treatment. *P*_2_ represents comparison between both groups after treatment.

**Table 6:** Random walking model of immune-inflammatory indices.

Index	Group	Maximum random fluctuation	Walking positive growth rate	Random fluctuation power law value	Improvement index	Comprehensive evaluation records	Ratio
ESR	Control group	1410	0.108	0.448 ± 0.099	0.226	6253	9.26
	Experimental group	2494	0.196	0.520 ± 0.126	0.362	6883	5.11

CRP	Control group	1915	0.137	0.494 ± 0.106	0.276	6933	7.28
	Experimental group	3236	0.239	0.566 ± 0.141	0.426	7603	4.18

IgA	Control group	666	0.058	0.400 ± 0.091	0.142	4676	17.33
	Experimental group	882	0.083	0.512 ± 0.135	0.184	4794	12.05

IgG	Control group	835	0.072	0.407 ± 0.095	0.179	4675	13.82
	Experimental group	979	0.092	0.463 ± 0.123	0.204	4800	10.86

C3	Control group	760	0.066	0.397 ± 0.091	0.163	4670	15.17
	Experimental group	1172	0.110	0.515 ± 0.128	0.244	4796	9.07

C4	Control group	1265	0.110	0.484 ± 0.127	0.270	4670	9.12
	Experimental group	1693	0.159	0.534 ± 0.121	0.353	4796	6.28

Note: ESR: erythrocyte sedimentation rate; CRP: C-reactive protein; IgA: immunoglobulin A; IgG: immunoglobulin G; C3: complement C3; C4: complement C4.

## Data Availability

The datasets generated for this study are available upon request from the corresponding authors.
